# Oral microbiota–immune crosstalk in the pathogenesis and progression of periodontitis: mechanisms and therapeutic potential

**DOI:** 10.3389/fmicb.2026.1953464

**Published:** 2026-10-15

**Authors:** Yelin Jin, Hua Xu

**Affiliations:** The First People’s Hospital of Wenling, Wenling, China

**Keywords:** disease progression, microbial dysbiosis, microbiota-immune crosstalk, oral microbiota, periodontitis, precision therapy

## Abstract

Periodontitis is a common oral inflammatory disease characterized by chronic inflammation and progressive destruction of the tooth-supporting tissues. Its initiation and progression are not driven solely by infection with specific periodontal pathogens, but rather arise from complex interactions among oral microbial dysbiosis, dysregulated host immune responses, and tissue remodeling. In recent years, oral microbiota–immune crosstalk has emerged as a central mechanistic link between microbial dysbiosis and periodontal tissue damage. Accumulating evidence indicates that oral microorganisms can modulate both innate and adaptive immune responses through microbial structural components, biofilm-associated factors, and metabolites. Conversely, persistent immune-inflammatory responses may reshape the composition and function of the oral microbiota by disrupting barrier integrity, altering the local metabolic milieu, and remodeling ecological niches, thereby establishing a potential self-reinforcing feedback loop. This review focuses on oral microbiota–immune crosstalk in periodontitis, summarizing the molecular mechanisms through which the microbiota regulates host immunity and, reciprocally, immune responses reshape the microbial ecosystem. Particular emphasis is placed on the dynamic changes in these interactions across disease-associated states, including inflammatory initiation, maintenance of chronic inflammation, periodontal tissue destruction, and establishment of a disease-associated microbial ecosystem. We further review the therapeutic and translational advances targeting the microbiota–immune network, including conventional periodontal therapy, biofilm modulation, probiotics, prebiotics and dietary interventions, adjunctive antimicrobial therapy, bacteriophage-based approaches, and engineered bacteria. Finally, we discuss future directions involving multi-omics integration, precision microbiota modulation, and individualized therapeutic strategies, with the aim of providing a conceptual framework and new avenues for the precision prevention and treatment of periodontitis and for the development of microbiota–immune-targeted therapies.

## 1 Introduction

Periodontitis is a prevalent oral disease characterized by chronic inflammation and progressive destruction of the tooth-supporting tissues and represents one of the leading causes of tooth loss in adults (Heitz-Mayfield, 2024; Kwon et al., 2021). Despite continuing advances in conventional periodontal therapy, antimicrobial interventions, and tissue-regenerative strategies, a substantial proportion of patients continue to experience inadequate treatment responses, disease recurrence, and ongoing periodontal tissue destruction (Wu et al., 2025; Wang X. et al., 2022; Huang et al., 2024). Traditionally, periodontitis was considered to be driven primarily by infection with specific periodontal pathogens and the ensuing inflammatory response (Wang X. et al., 2022). Accumulating evidence, however, supports a more complex disease model in which oral microbial dysbiosis, aberrant host immune responses, and pathological tissue remodeling collectively contribute to the initiation and progression of chronic periodontal inflammation (Lv M. et al., 2026).

The oral microbiota constitutes one of the major microbial ecosystems of the human body and plays an essential role in maintaining oral ecological stability, regulating mucosal barrier function, and shaping host immune homeostasis (Santacroce et al., 2023). Marked remodeling of the oral microbial ecosystem has been observed in patients with periodontitis. This remodeling is characterized not only by the enrichment of specific potentially pathogenic microorganisms but also by alterations in community structure, metabolic function, and biofilm ecology (Wang Y. et al., 2024; Cui et al., 2025). Periodontitis-associated microorganisms, including *Porphyromonas gingivalis, Tannerella forsythia*, and *Treponema denticola*, contribute to the establishment of a periodontal inflammatory microenvironment by forming complex polymicrobial biofilms, modifying local ecological conditions, and releasing a wide range of microbe-associated signals (Ursu et al., 2022; Potocnik Rebersak and Schara, 2025).

The immune system is a critical regulatory network responsible for maintaining periodontal tissue homeostasis and responding to microbial challenges. During the development and progression of periodontitis, oral microorganisms and host immunity engage in complex bidirectional interactions. Microbial-derived signals, including cell-wall components, virulence factors, and metabolic products, are sensed by pattern-recognition receptors and related signaling systems, such as Toll-like receptors, thereby activating innate immune responses and reshaping the functions of macrophages, neutrophils, and dendritic cells (Kumar and Stewart Iv, 2024; Li Y. et al., 2025; Cheok et al., 2022). Conversely, sustained immune activation can alter the local ecological niche through the release of inflammatory mediators, increased oxidative stress, disruption of the periodontal epithelial barrier, and changes in nutrient availability, thereby influencing microbial colonization, community organization, and metabolic activity (Baima et al., 2025a; Romandini et al., 2026; Han et al., 2023). These reciprocal interactions may establish a self-reinforcing cycle in which microbial dysbiosis amplifies inflammation, while the inflammatory microenvironment further favors the persistence of a disease-associated microbiota.

In recent years, several reviews have addressed complementary aspects of microbiota–host interactions in periodontal and oral diseases. Lv M. et al. (2026) discussed host–microbiome–immune disequilibrium across oral diseases and its implications for precision therapeutics, providing a broad framework extending beyond periodontitis. Cui et al. (2025) focused on microbial dysbiosis, immune responses, and therapeutic implications in periodontitis and peri-implantitis, whereas Han and Ding (2025) emphasized methodological advances and challenges in periodontal microbiome research, including study design, analytical strategies, and emerging technologies. Yekani et al. (2025), in contrast, primarily summarized the microbiological and molecular mechanisms underlying infection-associated periodontal inflammation. Building on these complementary perspectives, the present review specifically integrates reciprocal microbiota-to-host and host-to-microbiota mechanisms within periodontitis and examines how these interactions evolve across a conceptual disease-phase framework. We further distinguish among different levels of supporting evidence, including human interventional studies, longitudinal and cross-sectional human studies, animal mechanistic studies, and *in vitro* experiments, and evaluate microbiota-modulating therapies according to their current evidence base and translational maturity. Accordingly, this review links bidirectional microbiota–immune crosstalk, disease-course dynamics, evidence hierarchy, and therapeutic readiness within a unified periodontitis-focused framework, covering conventional periodontal therapy and biofilm control, probiotics, prebiotics and dietary interventions, adjunctive antimicrobial therapy, bacteriophage-based approaches, and engineered bacteria.

This article is positioned as a narrative review aimed at integrating mechanistic evidence on oral microbiota–immune crosstalk in periodontitis and summarizing recent advances in related therapeutic research, rather than providing a systematic review or meta-analysis. To improve the transparency of literature selection, we primarily searched the PubMed and Web of Science databases from inception through July 2026. Search terms included periodontitis, oral microbiota, oral microbiome, microbial dysbiosis, biofilm, host immunity, immune response, inflammation, microbial metabolites, microbiota–immune crosstalk, probiotics, bacteriophage, and microbiome-targeted therapy, which were combined according to the specific topic under investigation. Priority was given to studies directly relevant to alterations in the oral microbial ecosystem in periodontitis, bidirectional microbiota–immune regulatory mechanisms, and microbiota-targeted therapies. Eligible evidence included *in vitro* mechanistic studies, animal experiments, human cross-sectional and longitudinal studies, clinical intervention studies, and multi-omics investigations. Representative high-quality reviews were also included to provide background context and conceptual frameworks, with preference given to recently published studies that directly supported the mechanistic or translational statements discussed. Because current mechanistic and translational evidence in periodontitis is predominantly centered on bacterial components, the oral microbiota–immune crosstalk discussed in this review focuses primarily on the oral bacterial microbiota and its associated structural components, metabolites, and biofilm ecology.

## 2 Oral microbial dysbiosis and disruption of immune homeostasis in periodontitis

The oral microbial ecosystem comprises a diverse array of components, including bacteria, fungi, viruses, and their associated metabolites (Baker et al., 2024). Given that direct mechanistic evidence and translational research on microbiota–immune crosstalk in periodontitis currently focus predominantly on the bacterial microbiota, this review primarily addresses the bidirectional interactions between oral bacterial communities—including their structural components, biofilm-associated factors, and metabolites—and host immunity. Under physiological conditions, oral bacterial communities and the host maintain a dynamic equilibrium. Commensal bacteria contribute to local ecological stability through microbial competition, nutrient regulation, and metabolic activity (Santacroce et al., 2023; Huo et al., 2026), whereas the host immune system constrains the excessive expansion of potentially harmful microorganisms through barrier defenses and tightly regulated immune responses (Wang et al., 2026).

In recent years, advances in 16S rRNA sequencing, metagenomics, and multi-omics technologies have enabled a more comprehensive characterization of the microbial alterations associated with periodontitis (Manoil et al., 2000). Compared with periodontal health, periodontitis is associated with substantial shifts in oral microbial community composition and enrichment of disease-associated taxa (Kang et al., 2026; Lee et al., 2026). Changes in microbial diversity, however, are not uniform across oral sampling compartments. In a within-subject 16S rRNA sequencing study comparing saliva, oral rinse, subgingival plaque, and gingival crevicular fluid (GCF), Shannon alpha diversity was higher in periodontitis in saliva, oral rinse, and subgingival plaque but not in GCF, whereas Bray–Curtis beta-diversity analysis demonstrated clear disease-associated restructuring of microbial communities, with the most distinct separation observed in GCF (Lee et al., 2026). These findings indicate that periodontitis-associated dysbiosis is more appropriately characterized by site-specific compositional restructuring rather than by a generalized reduction in microbial diversity.

Earlier studies emphasized the roles of specific periodontal pathogens in disease development, particularly members of the so-called red complex, including *Porphyromonas gingivalis, Tannerella forsythia*, and *Treponema denticola* (Amani et al., 2026). More recent ecological models, however, suggest that periodontitis is not driven directly by a single pathogen, but is better understood as a dysbiosis-driven disease, in which community-wide alterations in microbial composition and function collectively contribute to disease development. Beyond compositional shifts, periodontal microbial dysbiosis also involves remodeling of biofilm architecture, altered intermicrobial interactions, and metabolic reprogramming, progressively creating a local environment that favors the persistence of inflammation (Chen et al., 2026; Lv W. et al., 2026).

In parallel with microbial dysbiosis, host immune homeostasis is profoundly disturbed during periodontitis. Periodontal tissues are continuously exposed to a complex microbial environment and therefore depend on a finely regulated immune network to maintain the balance between antimicrobial defense and immune tolerance (Antoniadou and Varzakas, 2026; Theofilou et al., 2026). In periodontitis, persistent microbial stimulation is closely associated with increased recruitment of inflammatory cells, amplification of pro-inflammatory responses, and impaired tissue repair (Martínez-García and Hernández-Lemus, 2025). Innate immune cells, including neutrophils, macrophages, and dendritic cells, contribute to the establishment of the inflammatory microenvironment during the early stages of disease (de Molon et al., 2026). As periodontitis progresses, imbalances among T-cell subsets, alterations in B-cell function, and dysregulated humoral immune responses are also associated with the persistence of chronic inflammation and progressive destruction of periodontal tissues (Wang P. et al., 2025; Xiang et al., 2025).

Importantly, oral microbial dysbiosis and disruption of immune homeostasis may jointly drive periodontitis progression through reciprocal interactions. Alterations in oral microbial communities may enhance host immune responses to dysbiotic microbial signals, whereas persistent inflammation can in turn modify the local periodontal ecological environment, thereby further influencing microbial colonization and community composition (Matsuoka et al., 2025; Demkovych et al., 2023).

## 3 Regulatory mechanisms of oral microbiota–immune crosstalk

Interactions between the oral microbiota and host immunity involve multilayered signal exchange, including the recognition of microbe-derived molecules, ecological regulation within polymicrobial biofilms, metabolite-mediated immune modulation, and the reciprocal reshaping of the microbial ecosystem by the inflammatory microenvironment. Distinct categories of microbial signals collectively contribute to the pathogenesis of periodontitis by modulating immune-cell functions, determining the magnitude and duration of inflammatory responses, and disrupting local tissue homeostasis. Within this bidirectional network, microbiota-mediated immune regulation represents a major initiating component, whereas the reciprocal effects of host immune responses on the oral ecological environment establish a feedback mechanism that promotes disease persistence and progression (see Figure 1).

**Figure 1:**
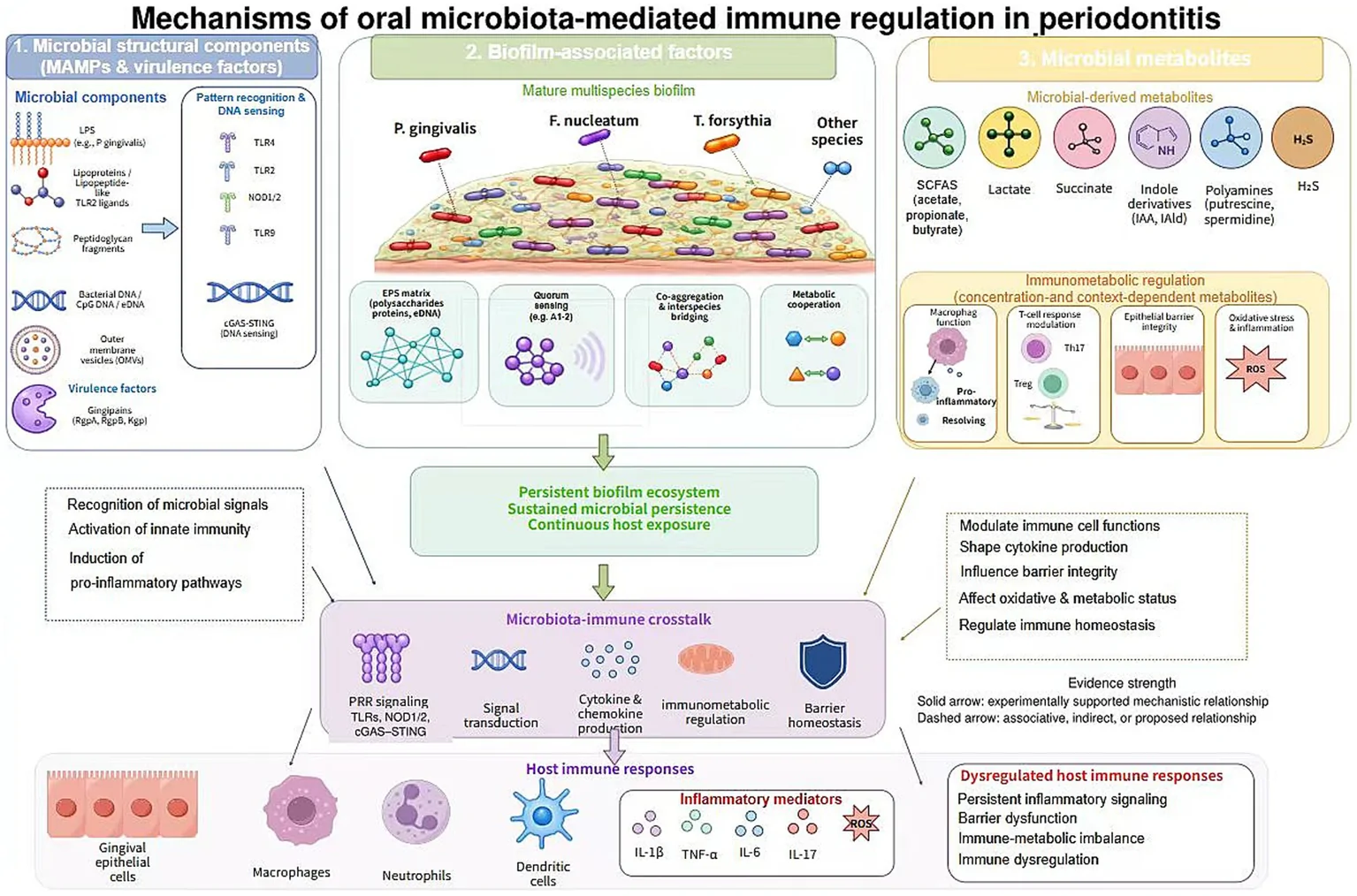
Mechanisms of oral microbiota-mediated immune regulation in periodontitis. Oral microorganisms regulate host immunity through three major categories of microbe-derived signals, including microbial structural components, biofilm-associated factors, and microbial metabolites. Structural components activate innate immune signaling through pattern-recognition receptors, whereas biofilm-associated factors promote microbial persistence and sustained host stimulation. Microbial metabolites further modulate immune cell function, epithelial barrier integrity, and inflammatory homeostasis in a context-dependent manner. These diverse microbial signals collectively establish oral microbiota–immune crosstalk, thereby influencing epithelial cells, macrophages, neutrophils, dendritic cells, adaptive immune responses, cytokine production, and barrier function. Persistent dysregulation of these interactions contributes to chronic inflammation, immune imbalance, and periodontal tissue destruction. Solid arrows indicate experimentally supported mechanistic relationships, whereas dashed arrows indicate associative, indirect, or proposed relationships. Solid arrows denote mechanistic support within the available experimental models and do not necessarily imply validation in humans. This figure illustrates a conceptual framework rather than a validated linear signaling pathway.

### 3.1 Oral microbiota-mediated regulation of host immunity

Oral microorganisms participate in the regulation of host immunity through multiple types of signals, including microbial structural components, biofilm-associated factors, and metabolic products. Rather than acting independently, these microbe-derived signals interact within the periodontal microenvironment to influence immune-cell activation, inflammatory response intensity, and local tissue homeostasis. Based on the currently available evidence, representative oral microbiota-associated signals involved in immune regulation during periodontitis can be broadly classified into three categories: microbial structural components, biofilm-associated factors, and microbial metabolites. Their principal immunoregulatory characteristics and supporting evidence are summarized in Table 1.

**Table 1:** Representative oral microbiota-associated signals involved in immune regulation during periodontitis.

Category	Microbiota-associated signal	Primary source	Principal target cells or processes	Immunoregulatory effects	Relevance to periodontitis	Main evidence types	References
Microbial structural components	Lipopolysaccharide (LPS)	Gram-negative periodontitis-associated bacteria, particularly *Porphyromonas gingivalis*	Macrophages, osteoclast precursors, and periodontal tissue cells	Promotes the expression of inflammatory mediators, including TNF, IL-1β, and IL-6, and enhances osteoclastogenesis through TLR-associated signaling and KAT6A-mediated epigenetic regulation	Amplifies gingival inflammation and osteoclast formation, thereby promoting alveolar bone resorption; its effects may vary according to age and host status	Cell-based experiments, patient single-cell transcriptomics, gene-knockout studies, and mouse periodontitis models	Demkovych et al. (2023); Huang et al. (2025)
Microbial structural components	Lipoproteins/lipopeptide-like TLR2 ligands	Bacterial lipoproteins or synthetic lipopeptide mimetics; periodontal pathogens may contain such molecules	Dendritic cells, gingival innate immune cells, and osteoclastogenic processes	Induce immunoregulatory factors such as BAFF, primarily through TLR2–MyD88–JNK/NF-κB signaling, and promote RANKL-associated osteoclastogenic responses	May induce gingival inflammation, inflammatory-cell infiltration, and alveolar bone loss; direct evidence for native lipoproteins derived from periodontal bacteria remains limited	Synthetic lipopeptide-based mouse models and mechanistic studies in dendritic cells	Akkaoui et al. (2021); Souza et al. (2020)
Microbial structural components	Peptidoglycan	Cell walls and peptidoglycan fragments derived from various oral and periodontal bacteria	Gingival epithelial barrier, intracellular pattern-recognition receptors such as NOD1, and periodontal ligament stem cells	*P. gingivalis* gingipains disrupt CXADR and facilitate peptidoglycan passage across the epithelium; peptidoglycan fragments activate NOD1–p38/MAPK signaling and inhibit osteogenic differentiation	Increases the access of bacterial components to subepithelial tissues and impairs the osteogenic and regenerative capacity of periodontal tissues	Three-dimensional gingival epithelial models, patient tissues, and mechanistic studies using human periodontal ligament stem cells	Im et al. (2020); Takeuchi et al. (2021)
Microbial structural components	Bacterial DNA/CpG DNA and extracellular DNA (eDNA)	Microbial eDNA released from periodontal biofilms; cfDNA in inflamed tissues also includes host-derived DNA	Gingival fibroblasts, TLR9-associated nucleic acid sensing, and the monocyte–macrophage system	Microbial eDNA–TLR9 signaling induces cathepsin K in fibroblasts, whereas cfDNA promotes proinflammatory responses and dysregulated macrophage polarization	May contribute to the early initiation of periodontitis and inflammatory bone loss; however, not all cfDNA should be considered bacterial DNA	Patient samples, cell-based experiments, single-cell transcriptomics, and mouse periodontitis models	He et al. (2022); Kondo et al. (2022)
Microbial structural components	Outer membrane vesicles (OMVs)	Gram-negative periodontal bacteria, including *P. gingivalis*	Gingival epithelial cells, periodontal ligament cells, and intracellular nucleic acid- and stress-sensing pathways	Deliver LPS, gingipains, DNA/RNA, and small RNAs, activating MAPK, NF-κB, STING, and related pathways and inducing inflammation and apoptosis	Facilitate the tissue dissemination of bacterial effector molecules and promote periodontal cell injury and alveolar bone resorption	Experiments using human gingival or periodontal ligament cells and rat periodontitis models	Huang et al. (2022); Uemura et al. (2022)
Microbial structural components	Gingipains (RgpA/RgpB/Kgp)	*P. gingivalis*	Macrophages, dendritic cells, gingival keratinocytes, and host proteins	Evade phagocytic clearance through proteolytic and nonproteolytic mechanisms; catalytically inactive RgpA can still induce proinflammatory mediators through EGFR–PI3K–AKT signaling	Promotes the persistence of *P. gingivalis* and creates a local environment favorable to inflammatory microbial communities, potentially contributing to periodontal tissue destruction	Mechanistic studies in human-derived cells, genetic deletion or inhibition experiments, and in vivo zebrafish infection models	Fan et al. (2023); Widziolek et al. (2025)
Biofilm-associated factors	Polymicrobial periodontal biofilms	Subgingival polymicrobial communities comprising commensals, bridging organisms, and periodontitis-associated bacteria	Microbial composition, spatial organization, metabolic interactions, and the host–microbe interface	Form stable and heterogeneous communities through interspecies adhesion, metabolic cross-feeding, and cooperative growth, with ecological restructuring occurring according to periodontal status	Community-wide dysbiosis, rather than a single microbial species, constitutes an important basis for persistent microbial stimulation in periodontitis	Cross-sectional sequencing of human subgingival microbiota and dynamic in vitro polymicrobial biofilm models	Ciaston et al. (2022); Ghesquière et al. (2023)
Biofilm-associated factors	Extracellular polymeric substances (EPS)	Bacteria and host inflammatory cells within periodontal biofilms; composed of polysaccharides, eDNA, proteins, and other components	Biofilm adhesion, matrix architecture, bacterial persistence, and resistance to clearance	Extracellular polysaccharides and eDNA enhance biofilm aggregation and stability; eDNA in patient-derived subgingival biofilms consists predominantly of host-derived components	Promotes bacterial colonization and long-term persistence; extracellular polysaccharides from *Aggregatibacter actinomycetemcomitans*can enhance experimental alveolar bone resorption	In situ imaging of patient subgingival biofilms, gene-deletion studies, and rat periodontal disease models	Iniesta et al. (2023); Slobodianyk-Kolomoiets et al. (2024)
Biofilm-associated factors	Quorum-sensing signals	AI-2 released by various periodontitis-associated bacteria and incompletely characterized AHL-like signals	Microbial communication, biofilm formation, virulence expression, and osteoclast differentiation	AI-2 regulates polymicrobial biofilms and promotes osteoclast differentiation, whereas quorum-quenching reduces the biomass of some patient-derived biofilms	May promote pathogenic biofilm maturation and alveolar bone loss and represents a potential target for antibiofilm intervention	Patient gingival crevicular fluid, in vitro polymicrobial biofilms, osteoclast assays, and mouse periodontitis models	Shanmugam et al. (2015); Li et al. (2024)
Biofilm-associated factors	*Fusobacterium nucleatum*-mediated microbial coaggregation	*F. nucleatum*	The RadD/CarRS regulatory network and coaggregation partners such as streptococci and *P. gingivalis*	Uses RadD to mediate interspecies adhesion under the regulation of CarRS and lysine metabolism; diffusible signals from *P. gingivalis*can enhance the expression of biofilm-associated genes in *F. nucleatum*	Functions as a bridging organism connecting early and late colonizers and promotes the maturation of polymicrobial biofilms	Transposon-based genetic screening, transcriptomics, in vitro coculture, and mouse infection models not specifically designed to model periodontitis	Parga et al. (2023); Wu et al. (2021)
Biofilm-associated factors	Keystone-pathogen activity of *P. gingivalis*	*P. gingivalis*	Microbial diversity, tryptophan–indole metabolism, virulence expression, and host inflammation	Alters microbial richness and composition in a strain-dependent manner; its tryptophan–indole pathway coordinates virulence, biofilm formation, and microbial dysbiosis	May act as an ecological modulator that promotes microbial dysbiosis and periodontal tissue destruction, although its effects are strain- and environment-dependent	Patient saliva and metabolomic analyses, in vitro mechanistic studies, and microbiome sequencing in mouse periodontitis models	Yamaguchi-Kuroda et al. (2023); Ding et al. (2025)
Microbial metabolites	Short-chain fatty acids (SCFAs: acetate, propionate, and butyrate)	Fermentation by oral anaerobes and selected commensal bacteria; production profiles vary among microbial taxa	Dendritic cells, macrophages, gingival fibroblasts, and oral epithelial cells	Exert concentration-, cell-, and source-dependent bidirectional effects; in the cited studies, butyrate inhibited dendritic-cell glycolysis and maturation and reduced NF-κB-associated proinflammatory mediators	May regulate periodontal immune homeostasis; however, these findings should not be generalized to suggest that all SCFAs or high concentrations of butyrate are protective	Mechanistic studies using human- and mouse-derived cells and mouse periodontitis models	Boyer et al. (2020); Wu et al. (2023)
Microbial metabolites	Lactate	Fermentation by oral microorganisms and glycolysis in local host cells; current studies cannot clearly distinguish their relative contributions	Macrophage lactylation and the local metabolic microenvironment	Influences macrophage inflammatory phenotypes through mechanisms such as protein lysine lactylation; salivary lactate may also serve as a metabolism-related diagnostic indicator	Associated with the inflammatory metabolic state of periodontitis, although causal evidence that microbially derived lactate directly drives periodontitis remains lacking	Patient tissues and saliva, macrophage and rat periodontitis studies, and diagnostic chip validation	Santos et al. (2022); Liu et al. (2024)
Microbial metabolites	Succinate	Oral microbiota and the host tricarboxylic acid cycle; periodontitis-associated bacteria may also produce succinate	SUCNR1, periodontal immune cells, osteoclasts, and gingival epithelial cells	Elevated succinate activates SUCNR1 and promotes inflammation and osteoclastogenesis; SUCNR1 antagonism or deficiency attenuates inflammation, dysbiosis, and bone loss	Represents a candidate pathogenic metabolic signal linking microbial metabolism, host inflammation, and alveolar bone resorption	Patient subgingival plaque and oral-rinse samples, bacterial culture, cell-based experiments, receptor-knockout studies, and mouse periodontitis models	Zhang et al. (2024); Guo et al. (2022)
Microbial metabolites	Indole metabolites, including indole-3-acetic acid (IAA) and indole-3-aldehyde (IAld)	Tryptophan metabolism by *P. gingivalis*and other oral microorganisms	Macrophages, gingival epithelial cells, and AhR-associated pathways	IAA promotes anti-inflammatory macrophage polarization through AhR/CYP1A1 signaling, whereas IAld maintains epithelial junctions and barrier integrity through AhR/Nrf2 signaling	Specific indole derivatives may reduce inflammation and bone resorption and preserve the gingival barrier; the effects of different indole metabolites should not be generalized	Targeted metabolomics, 16S rRNA sequencing, human-derived cell experiments, and mouse periodontitis models	Ishihara et al. (2025); Cheng et al. (2026)
Microbial metabolites	Polyamines, including putrescine and spermidine	Ornithine and amino acid metabolism by oral bacteria such as *F. nucleatum*; periodontal tissues may also contain host-derived polyamines	Polymicrobial biofilm metabolism, oxidative stress in periodontal cells, and inflammatory responses	*F. nucleatum* increases putrescine and cadaverine production during coculture; excessive polyamines can enhance oxidative stress and proinflammatory responses, whereas polyamine sequestration suppresses these effects	May link metabolic interactions within the microbiota to periodontal inflammation; different polyamines and concentrations may exert distinct effects	In vitro polymicrobial metabolomics, patient gingival crevicular fluid, cell-based experiments, and animal periodontitis models	Wu et al. (2026); Sakanaka et al. (2022)
Microbial metabolites	Hydrogen sulfide (H₂S)	Sulfur-containing amino acid metabolism by periodontal anaerobes; host enzymatic systems can also generate H₂S	Periodontal ligament cells, autophagy and oxidative-stress pathways, and the local inflammatory environment	High concentrations or exogenous slow-release H₂S reduce cell viability, enhance inflammatory mediator production, and induce autophagy; the effects are concentration- and source-dependent	Elevated local H₂S is associated with periodontitis and increased volatile sulfur compounds and may aggravate experimental inflammation and bone loss	Human periodontal ligament cell experiments, rat periodontitis models, and human cross-sectional studies	Wang et al. (2025); Ni and Hua (2021)
Microbial metabolites	Ammonia/ammonium and alkali-generating metabolism	Bacterial urease and arginine deiminase systems in the oral microbiota; these processes should not be described solely as protein metabolism	Local pH, calculus mineralization, and microbial ecology; no clearly established periodontal immune-cell target	Ammonia/ammonium production increases local pH and alters mineral saturation and microbial competitive relationships	May indirectly influence periodontal status by promoting calculus formation and modifying oral ecology; direct causal evidence involving periodontal immunity remains unavailable	Human cross-sectional periodontal and calculus studies and mechanistic oral microbiome metatranscriptomic research providing indirect evidence	Lee et al. (2023); D’souza et al. (2023)

#### 3.1.1 Immune recognition and inflammatory initiation mediated by microbial structural components

Microbe-associated molecular patterns (MAMPs) represent a major source of microbial signals recognized by the host in periodontal tissues. Alterations in microbial community composition and biofilm architecture may increase periodontal exposure to these signals and contribute to local immune activation through pattern-recognition receptor–dependent pathways.

Lipopolysaccharide (LPS) is among the most extensively studied microbial structural components. LPS derived from Gram-negative periodontitis-associated bacteria, including *Porphyromonas gingivalis*, can promote the expression of inflammatory mediators such as TNF, IL-1β, and IL-6 and influence osteoclastogenesis and periodontal inflammation through Toll-like receptor TLR-related signaling pathways (Huang et al., 2025; Akkaoui et al., 2021). Lipoproteins and lipopeptide-like TLR2 ligands can regulate dendritic-cell responses and osteoclast-associated processes through a signaling axis involving TLR2, myeloid differentiation primary response 88 (MyD88), JNK, and NF-κB (Souza et al., 2020; Im et al., 2020). Peptidoglycan may gain access to periodontal tissues following disruption of the gingival barrier and subsequently affect periodontal cell function through pathways such as NOD1–p38/MAPK signaling (Takeuchi et al., 2021; He et al., 2022). Microbial extracellular DNA may also contribute to experimental periodontitis and alveolar bone loss through TLR9-associated nucleic acid sensing and tissue-degradative pathways (Kondo et al., 2022; Huang et al., 2022).

Outer membrane vesicles (OMVs) can transport multiple effector molecules, including LPS, gingipains, and nucleic acids. Cell-based and animal studies have shown that *P. gingivalis-derived* OMVs can activate MAPK, NF-κB, and STING signaling, thereby inducing inflammatory responses and cellular injury (Uemura et al., 2022; Fan et al., 2023). Gingipains may additionally impair macrophage phagocytic clearance and modulate host inflammatory signaling (Widziolek et al., 2025; Ciaston et al., 2022). Nevertheless, these mechanisms are still supported predominantly by preclinical evidence, and their relative contributions to human periodontitis remain to be established.

#### 3.1.2 Periodontal biofilms in microbial ecological stability and immune regulation

A defining feature of periodontitis is the formation of complex multispecies biofilms rather than infection by planktonic bacteria alone. Subgingival microorganisms can establish spatially and functionally heterogeneous communities through interspecies adhesion, metabolic complementation, and cooperative growth. Both human sequencing studies and *in vitro* models suggest that periodontitis-associated microbial changes are better characterized as community-wide ecological restructuring than as the independent expansion of a single bacterial species (Ghesquière et al., 2023; Iniesta et al., 2023).

Components of EPS, including polysaccharides, extracellular DNA, and proteins, can enhance biofilm aggregation, structural stability, and persistent microbial colonization. Some experimental studies have further linked these components to alveolar bone resorption (Slobodianyk-Kolomoiets et al., 2024; Shanmugam et al., 2015). Quorum-sensing signals can regulate multispecies biofilm formation, virulence expression, and osteoclast-related processes, whereas disruption of quorum sensing has been shown to reduce the formation of certain periodontal biofilms (Li et al., 2024; Parga et al., 2023).

Interbacterial coaggregation also contributes to biofilm maturation. *Fusobacterium nucleatum* can mediate interspecies adhesion through regulatory networks involving RadD and CarRS and may function as a bridging organism connecting microorganisms that colonize at different stages of biofilm development. Signals derived from *P. gingivalis* can also enhance the expression of biofilm-associated genes in *F. nucleatum* (Wu et al., 2021; Yamaguchi-Kuroda et al., 2023). In addition, *P. gingivalis* may contribute to microbial dysbiosis by modulating microbial metabolism, virulence, and community composition, although these effects appear to be strain- and context-dependent (Ding et al., 2025; Boyer et al., 2020). At present, much of the mechanistic evidence in this area still derives from *in vitro* and animal studies, and the dynamic roles of these interactions within human periodontal biofilms require further validation.

#### 3.1.3 Immunometabolic regulation mediated by microbial metabolites

Microbial metabolites constitute an important mechanistic link between the functional state of the oral microbiota and host immunity, and their biological effects are often dependent on concentration, source, cell type, and the surrounding microenvironment. SCFAs can modulate the functions of dendritic cells, macrophages, gingival fibroblasts, and epithelial cells. Some studies have shown that butyrate and propionate can suppress specific inflammatory responses; however, their effects are strongly dose- and cell-context dependent, indicating that SCFAs should not be uniformly regarded as protective metabolites (Wu et al., 2023; Santos et al., 2022).

Lactate can be generated by both microbial fermentation and glycolysis in host cells, and the relative contributions of these two sources remain difficult to distinguish accurately. Experimental evidence suggests that lactate-associated protein lactylation can modulate inflammatory phenotypes in macrophages; however, current evidence remains insufficient to establish that microbiota-derived lactate directly drives periodontitis (Liu et al., 2024; Zhang J. et al., 2024). Succinate, in contrast, can promote inflammation and osteoclastogenesis through SUCNR1, whereas blockade of this pathway has been shown to attenuate experimental inflammation, microbial dysbiosis, and bone loss (Guo et al., 2022; Ishihara et al., 2025).

Tryptophan-derived indole metabolites may also participate in periodontal immune regulation. Indole-3-acetic acid can influence macrophage polarization through the AhR/CYP1A1 pathway, whereas indole-3-aldehyde may preserve gingival epithelial barrier integrity through AhR/Nrf2 signaling (Cheng et al., 2026; Wu et al., 2026). In addition, polyamines, hydrogen sulfide, and urea/arginine-related metabolism may affect the periodontal microenvironment by modulating oxidative stress, local pH, and intermicrobial competition (Sakanaka et al., 2022; Wang S. et al., 2025; Ni and Hua, 2021; Lee et al., 2023; D’souza et al., 2023; Mann et al., 2024). Nevertheless, compared with microbial structural components, direct mechanistic evidence for several metabolites in human periodontal tissues remains limited. Future studies integrating targeted metabolomics, functional experiments, and longitudinal clinical investigations will therefore be required to clarify their sources, temporal dynamics, and causal roles.

### 3.2 Reciprocal effects of immune-inflammatory responses on Oral microbial remodeling

Current evidence indicates that the periodontal microbiota and host inflammation interact bidirectionally, rather than through a simple unidirectional “microbial stimulation–host inflammation” pathway. In a human experimental gingivitis model, cessation of oral hygiene induced coordinated changes in plaque microbial composition, metabolic profiles, and salivary cytokines within 24–72 h, with the taxonomic and functional characteristics of the microbiota progressively shifting toward a periodontitis-associated state (Huang et al., 2021). Because this study primarily captured temporal associations among these parameters, it supports the synchronous evolution of inflammation and microbial community changes but does not, by itself, establish host inflammation as the direct cause of microbial alterations. More direct evidence has been obtained from CD69-deficient mice, in which CD69 deficiency enhanced IL-17A responses in gingival CD4^+^ T cells and aggravated alveolar bone loss while markedly altering the composition of ligature-associated biofilms (Saavedra et al., 2025).

Inflammation-induced changes in the local nutrient environment may represent an important link between host responses and microbial community remodeling. At periodontal sites undergoing clinical progression, gingival crevicular fluid proteomic profiles are enriched for processes related to catabolism, lipid metabolism, and hemoglobin- and haptoglobin-binding, suggesting that active inflammation is accompanied by altered availability of host-derived substrates within periodontal pockets (Torres et al., 2023). *Porphyromonas gingivalis* preferentially acquires dipeptides through the proton-dependent oligopeptide transporter Pot, and loss of Pot markedly impairs bacterial growth, indicating that peptide substrates represent an important nutrient source for this organism (Ohara-Nemoto et al., 2020). In patient-derived periodontal and peri-implant biofilms, *P. gingivalis* gingipains and the KLIKK proteases of *Tannerella forsythia* are expressed *in vivo*, while the corresponding bacterial loads and local levels of IL-1β and IL-8 are associated with disease severity (Eckert et al., 2018).

Neutrophil-mediated oxidative stress and neutrophil extracellular traps (NETs) constitute another potential inflammatory amplification pathway. In experimental periodontitis, fibrin deposition promotes mitochondrial reactive oxygen species production in neutrophils and induces the formation of NETs enriched in mitochondrial DNA; scavenging mitochondrial reactive oxygen species reduces NET formation and periodontal inflammation (Chen et al., 2025). Human studies further indicate that NETs and extracellular histones can enhance IL-17/Th17 responses and promote alveolar bone destruction, while NET-associated complexes are markedly increased in both local lesions and peripheral blood from patients with severe periodontitis (Kim et al., 2023). Bacteria within dental plaque biofilms can also render NET-derived DNA resistant to DNase-mediated degradation, thereby facilitating apatite nucleation and calculus formation, suggesting that microorganisms can in turn influence the persistence and stability of NETs within the local microenvironment (Wan et al., 2024).

Immune-mediated disruption of the gingival epithelial barrier may further increase direct contact between host tissues and microorganisms. In human gingival epithelial models, neutrophil elastase cleaves junctional proteins including desmoglein-1, occludin, and E-cadherin, thereby increasing epithelial permeability and facilitating the penetration of periodontal pathogens. In mouse models, inhibition of neutrophil elastase also reduces gingival *Il1b* and *Il6* expression and attenuates alveolar bone loss (Hiyoshi et al., 2022). These findings support a role for neutrophil-derived proteases in gingival epithelial barrier disruption; however, their direct effects on the composition of the periodontal microbiota remain to be determined.

Complement activation represents another host-derived mechanism capable of amplifying periodontal inflammation. A phase IIa clinical trial showed that local administration of the C3 inhibitor AMY-101 significantly reduced gingival inflammation scores, bleeding on probing, and gingival crevicular fluid levels of MMP-8 and MMP-9 (Hasturk et al., 2021), supporting the involvement of complement signaling in periodontal inflammation and tissue degradation. Notably, however, the study did not directly assess restoration of gingival epithelial barrier integrity or perform microbiome sequencing. Therefore, it cannot yet be concluded that C3 inhibition restores gingival barrier function or periodontal microbial homeostasis.

Immunometabolic reprogramming may also contribute to remodeling of the periodontal microenvironment by altering local nutrient availability, redox balance, and energy metabolism. In a mouse model of periodontitis, nanoformulated quercetin suppressed glycolysis in inflammatory macrophages, enhanced oxidative phosphorylation, and reduced periodontal tissue levels of IL-1β, TNF-α, and reactive oxygen species, together with attenuation of alveolar bone loss (Zhang Y. et al., 2024). Another study showed that aspirin-loaded extracellular vesicles similarly inhibited macrophage glycolysis, promoted oxidative phosphorylation, and alleviated experimental periodontitis (Shi et al., 2023). However, neither study concurrently assessed the composition of the periodontal microbiota. Thus, current evidence supports a role for immunometabolic status in regulating periodontal inflammation, but does not yet demonstrate that immunometabolic alterations directly select for or enrich specific periodontal microbial communities.

### 3.3 Modulation of oral microbiota–immune crosstalk by host and environmental factors

Oral microbiota–immune crosstalk does not occur in isolation; rather, its magnitude and direction are jointly shaped by host characteristics and environmental exposures. Smoking, diabetes or hyperglycemia, age, oral hygiene status, dietary patterns, medication use, and other systemic factors may alter the composition and function of the oral microbiota by modifying the local nutrient milieu, oxidative stress, barrier integrity, and host immune responses. Long-term differences in exposure associated with geography, lifestyle, and population background may also partly account for the heterogeneity observed across periodontal microbiome studies.

Longitudinal studies have shown that smokers exhibit distinct subgingival microbial community structures even at comparable probing depths, and some ecological shifts may precede overt clinical disease progression. Smoking is also associated with altered local periodontal cytokine responses and differences in inflammatory resolution after treatment (Tamashiro et al., 2023; Hao et al., 2023). Diabetes and hyperglycemia likewise affect both the microbial and immune environments. Population-based studies have identified distinct subgingival microbial profiles in patients with diabetes and periodontitis, while levels of advanced glycation end products are associated with specific microbial taxa and clinical disease parameters. Single-cell and experimental studies further indicate that hyperglycemia is accompanied by expansion of γδ T cells, reduction in regulatory T cells, and an increase in pro-inflammatory neutrophil populations, suggesting that metabolic dysregulation can reshape the periodontal microenvironment through host immune mechanisms (Chen J. et al., 2024; Alghamdi et al., 2025).

Age represents another important host-modifying factor. Experimental periodontitis studies have shown that aged animals exhibit greater alveolar bone loss, increased cellular senescence, and enhanced expression of inflammation-related genes, together with alterations in the oral microbiota. These findings suggest that immunosenescence and microbial ecological changes may act in concert to increase periodontal susceptibility in older individuals (Unlu et al., 2026). Oral hygiene can directly alter both biofilm ecology and host inflammatory status. Experimental gingivitis studies have demonstrated progressive remodeling of the dental plaque microbiota following withdrawal of oral hygiene, whereas restoration of oral hygiene is accompanied by a gradual return of the microbial community toward its baseline state (Hall et al., 2023). Dietary factors may also contribute to this regulatory network. Intermittent fasting has been reported to reduce systemic and periodontal inflammation while exerting relatively limited effects on overall subgingival microbial diversity, suggesting that dietary modulation of host inflammation and microbial ecology may not necessarily occur in parallel (Lira-Junior et al., 2024).

Medication use is another important confounding factor that should be considered when interpreting differences in oral microbiome profiles. Studies in older adults have shown that commonly used medications, including statins, proton pump inhibitors, and bisphosphonates, are associated with differences in salivary microbial community composition, with the explanatory effects of some medications exceeding those of age, sex, or oral health status itself (Hisamatsu et al., 2025). In addition, various systemic inflammatory diseases may interact with periodontitis through shared abnormalities in innate immunity, inflammatory mediators, and metabolic environments, further adding complexity to oral microbiota–immune crosstalk (Wu Q. et al., 2024).

## 4 Dynamic disease-phase framework of oral microbiota–immune crosstalk during periodontitis progression

Accumulating evidence suggests that oral microbiota–immune crosstalk operates throughout the development and progression of periodontitis and evolves dynamically in parallel with changes in microbial communities, host immune status, and the local tissue microenvironment. To facilitate integration of the available evidence, we conceptually organize these changes into several disease-related phases, including inflammatory initiation, maintenance of chronic inflammation, tissue destruction, and persistence of advanced disease, based on findings from human observational and longitudinal studies as well as experimental models. Importantly, this framework is intended to describe potential temporal and pathobiological changes rather than a fixed biological staging system that has been validated in human populations, and it should not be conflated with the formal Stage I–IV clinical classification of periodontitis. At present, most mechanistic evidence with temporal resolution derives from animal models, whereas a substantial proportion of human studies remain cross-sectional or based on comparisons between established disease states (see Figure 2).

**Figure 2:**
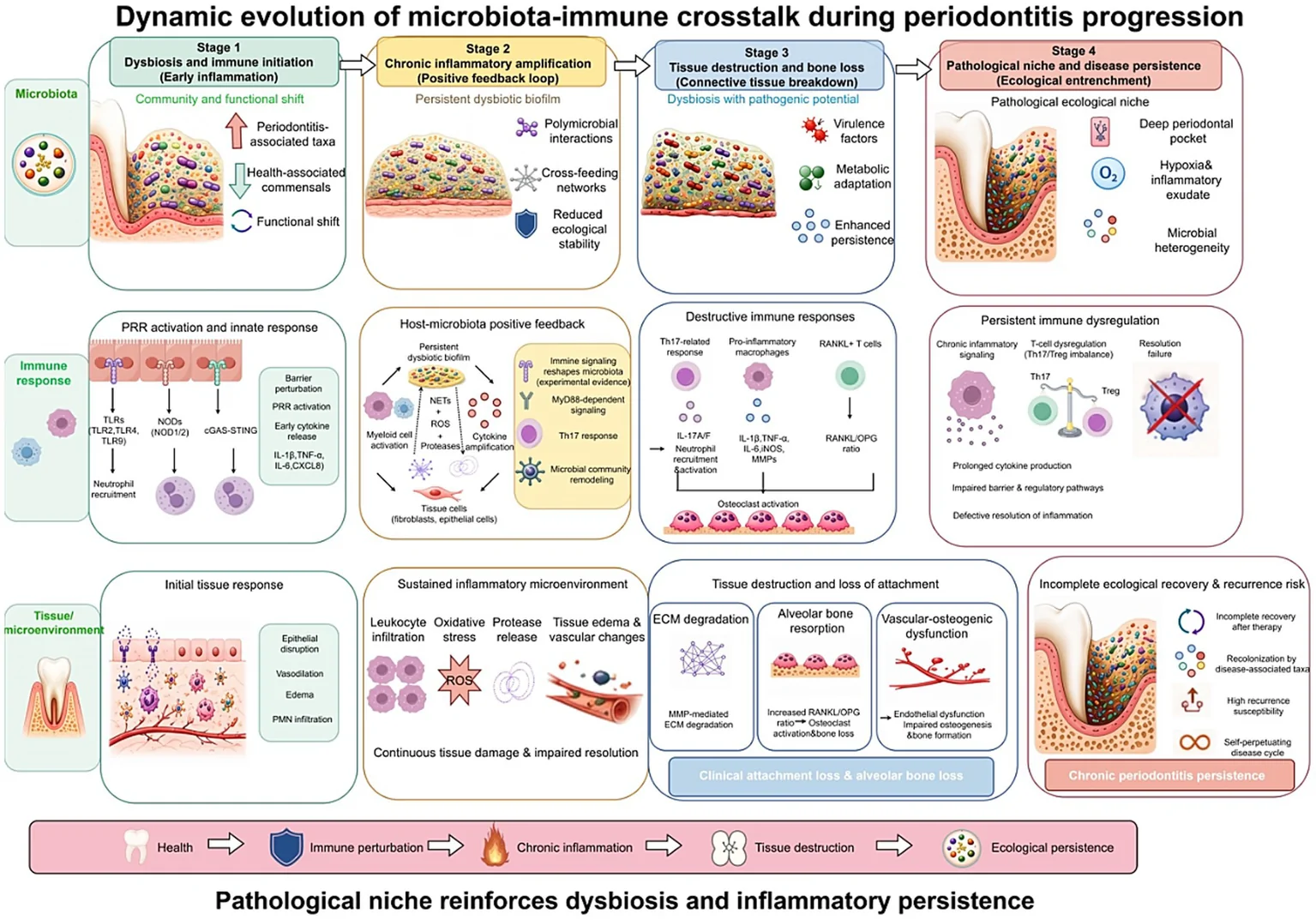
Dynamic evolution of microbiota–immune crosstalk during periodontitis progression. This figure summarizes the dynamic disease-phase evolution of oral microbiota–immune crosstalk during periodontitis progression. The proposed framework comprises four interconnected phases: (1) dysbiosis initiation, characterized by microbial community shifts and disruption of immune homeostasis; (2) chronic inflammatory amplification, involving persistent biofilm formation, immune activation, and positive feedback between dysbiosis and inflammation; (3) tissue destruction, marked by extracellular matrix degradation, alveolar bone resorption, vascular dysfunction, and immune-mediated tissue remodeling; and (4) pathological niche establishment, in which altered ecological conditions promote microbial persistence, inflammatory maintenance, and disease recurrence. Rather than representing a strictly linear sequence, the figure illustrates a dynamic positive-feedback network in which microbial dysbiosis, immune dysregulation, and tissue remodeling continuously reinforce one another during disease progression.

### 4.1 Inflammation-initiation–related changes: from microbial ecological shifts to disruption of immune homeostasis

During inflammation-initiation–related changes, shifts in the overall composition and ecological function of the subgingival microbial community appear to be more prominent than the effects of any single periodontal pathogen. Studies based on 16S rRNA sequencing have shown that periodontal health and different stages of periodontitis are associated with distinct, although partially overlapping, subgingival microbial profiles. With increasing disease severity, periodontitis-associated genera such as *Porphyromonas, Tannerella, Filifactor,* and *Fretibacterium* become increasingly prominent, whereas health-associated genera including Streptococcus and Haemophilus are relatively enriched in periodontally healthy individuals (Liu et al., 2026). A study analyzing 506 subgingival samples from four countries further identified periodontal diagnostic status as the principal variable explaining differences in subgingival microbial composition, while the country of origin also exerted a significant effect. These findings highlight geography, lifestyle, and population background as important potential confounders in periodontal microbiome research (Arredondo et al., 2023).

Disease-associated alterations are not limited to taxonomic abundance but also involve changes in microbial functional states. Paired metagenomic and metatranscriptomic analyses have demonstrated that periodontitis is associated with species-specific changes in gene expression within the subgingival microbiota, together with reduced activity of carbohydrate metabolic pathways, indicating that the mere presence or absence of particular bacteria does not adequately reflect their functional activity or pathogenic potential (Belstrøm et al., 2021). Accordingly, this phase may be more appropriately characterized as a coordinated shift in microbial composition, gene expression, and metabolic function toward an inflammation-adapted ecological state, rather than simply as an increase in the abundance of periodontal pathogens.

The host barrier exhibits cell type–specific responses to these microbial ecological changes. An integrated single-cell and spatial analysis of 34 samples comprising 105,918 cells revealed altered differentiation states of sulcular and junctional epithelial keratinocytes in periodontitis, accompanied by enhanced expression of inflammatory effector molecules. Intracellular or membrane-associated bacterial signals were also associated with pro-inflammatory phenotypes in keratinocytes (Easter et al., 2024). Spatial transcriptomic analysis of human gingival tissues further demonstrated increased JAK–STAT and NF-κB signaling in vascular endothelial cells from periodontitis lesions, with inflammation-related genes such as IFI16 displaying distinct spatial patterns (Shen et al., 2024). However, because both human studies primarily compared established periodontitis tissues with healthy controls, they identify disease-associated cellular-state differences but cannot independently establish whether these alterations precede clinically detectable disease.

Animal studies provide more direct evidence regarding temporal sequence. Single-cell time-course analysis in a murine ligature-induced periodontitis model identified an early-emerging fibroblast population specialized in directing leukocyte migration, characterized by chemokine expression and association with neutrophil recruitment. Once chronic inflammation was established, this fibroblast–neutrophil network became further linked to ILC3-derived IL-17 and alveolar bone resorption (Kondo et al., 2023). A standardized reanalysis of nine murine ligature-induced periodontitis datasets similarly found that marked alterations in microbial community structure generally became apparent from approximately day 5 after ligature placement; however, the source study itself also exerted a significant effect on microbial composition (Arce et al., 2022).

### 4.2 Maintenance of chronic inflammation: immune dysregulation and stabilization of a disease-associated microbial ecosystem

During the chronic inflammation–maintenance phase, periodontitis is characterized by a persistent inflammatory network involving stromal cells, myeloid cells, and adaptive immune cells, rather than by a transient antimicrobial response alone. Single-cell atlases of human gingiva have shown that epithelial and stromal cells in periodontitis exhibit enhanced inflammatory programs and neutrophil-recruiting signatures, accompanied by increased numbers of immune cells such as neutrophils and plasma cells (Williams et al., 2021). These findings suggest that gingival stromal cells may actively participate in local leukocyte recruitment and regulation of the mucosal immune environment.

Macrophages display marked heterogeneity during this phase. Single-cell analyses have shown that macrophages in periodontitis co-express markers associated with both pro-inflammatory and anti-inflammatory states and exhibit transcriptional features linked to regulatory programs involving factors such as RELA (RELA proto-oncogene, NF-κB subunit p65) (Agrafioti et al., 2022). Adaptive and innate-like lymphocytes also contribute to the maintenance of inflammation. In patients with periodontitis, mucosal-associated invariant T cells exhibit increased expression of activation markers such as CD69, enhanced production of IL-17 and TNF-α, and expression of CCR6 and CXCR6, which may facilitate their recruitment to inflamed gingival tissues (Kim et al., 2022).

Causal evidence that host immune status can reciprocally shape the microbiota has been derived primarily from animal studies. In a *P. gingivalis*-induced model, MyD88 deficiency reduced Th17 responses, the RANKL/OPG ratio, osteoclast formation, and alveolar bone loss, while simultaneously shifting the subgingival microbial community from one dominated by Gram-negative anaerobes toward greater representation of Gram-positive and potentially beneficial bacteria. These findings directly support a role for host immune signaling in microbial ecological selection within this experimental model (Hsiao et al., 2024).

Persistent neutrophil activation is another important component of chronic periodontal inflammation. Single-cell analysis of human gingiva identified a NETs-related neutrophil subpopulation, while *in vivo* inhibition of NET formation reduced gingival inflammatory infiltration and alveolar bone resorption (Qiu et al., 2025). Cell–cell communication analysis further suggested that gingival fibroblasts may promote NET formation through the MIF–CD74/CXCR4 axis (Qiu et al., 2025). However, current evidence does not establish that NETs directly select for or enrich specific periodontal bacterial species.

Th17-related responses likewise exhibit substantial functional plasticity and should not be regarded as uniformly pathogenic. In mice, a subset of Th17 cells can undergo IL-6-dependent conversion into T follicular helper-like cells within draining lymph nodes. Blocking this transition reduces oral IgG levels, increases the overall commensal bacterial burden, and exacerbates alveolar bone loss, indicating that certain Th17-derived populations may also exert protective effects by contributing to microbial control (McClure et al., 2024). More recent spatial multi-omics studies in humans have further shown that even the healthy tooth–gingiva interface contains a neutrophil layer and discrete aggregates of antigen-presenting cells and lymphocytes, whereas these regions expand in periodontitis and develop into immature tertiary lymphoid-like structures (Theofilou et al., 2026b).

### 4.3 Tissue destruction: inflammation-driven matrix remodeling and alveolar bone resorption

The tissue destruction–related phase is typically characterized by extracellular matrix degradation and loss of periodontal attachment structures. A study involving 190 participants showed that a biomarker panel comprising MMP-9, S100A8, α1-acid glycoprotein, and pyruvate kinase could effectively discriminate periodontal health, gingivitis, and periodontitis, supporting the clinical association of MMP-9 with periodontal disease status (Grant et al., 2022). However, this study alone does not establish MMP-9 as the sole driver of collagen degradation.

More direct mechanistic evidence indicates that PIEZO1 expression is increased in periodontal tissues from patients with periodontitis. PIEZO1 promotes a pro-inflammatory macrophage phenotype and the production of inflammatory mediators, which in turn induces MMP-8 and MMP-13 expression and promotes degradation of type I and type III collagen, thereby establishing a macrophage mechanosensing–MMP–collagen degradation axis (Zhao et al., 2023). In addition, *P. gingivalis*-derived outer membrane vesicles can be internalized by vascular endothelial cells and activate the cGAS–STING–TBK1 pathway, resulting in endothelial dysfunction and subsequent impairment of the migration and early osteogenic differentiation of osteogenic-lineage cells. Consistently, animal experiments have shown reduced numbers of Runx2-positive cells and increased alveolar bone loss (Chen Z. et al., 2024).

Alveolar bone resorption is closely linked to amplified inflammatory signaling and dysregulated osteoclastogenesis. In murine periodontitis, MyD88 deficiency reduces the RANKL/OPG ratio and the number of TRAP-positive osteoclasts, supporting a mechanism by which microbial sensing signals promote bone resorption through the immune–RANKL axis (Hsiao et al., 2024). Another study integrating human gingival crevicular fluid analyses with murine genetic models showed that loss of type I interferon signaling in myeloid cells enhances the IL-17–neutrophil axis while directly relieving suppression of osteoclast differentiation in monocytes (Zhang et al., 2025).

Osteoclast-intrinsic pathways also contribute to periodontal bone resorption. FoxO1-positive osteoclasts are enriched in alveolar bone during experimental periodontitis, whereas FoxO1 knockdown suppresses NF-κB and NOD-like receptor family pyrin domain containing 3 (NLRP3) inflammasome activation and thereby inhibits RANKL-induced osteoclast formation and maturation (Wang Z. et al., 2024). Inhibition of cathepsin K can additionally promote platelet-derived growth factor-BB (PDGF-BB) secretion from preosteoclasts, enhance type H vessel formation through the PDGF-BB/platelet-derived growth factor receptor-β (PDGFR-β) axis, and attenuate alveolar bone resorption in mice, indicating that coupling between angiogenesis and bone remodeling also contributes to periodontal bone homeostasis (Zhou et al., 2024).

Immune-modulatory interventions provide further causal support for these mechanisms. Local administration of CD73-enriched extracellular vesicles derived from induced regulatory T cells reduces activated IL-17A^+^ and RANKL^+^ CD4^+^ T cells through adenosine-related mechanisms, decreases osteoclast numbers, and attenuates experimental alveolar bone loss (Rojas et al., 2025).

### 4.4 Advanced disease: establishment of a pathological microbial ecosystem and disease persistence

In advanced or severe periodontitis, deepening periodontal pockets and progressive attachment loss create a spatial environment that differs substantially from the healthy gingival sulcus. Deep amplicon sequencing of 121 individual periodontal pockets from nine patients identified three distinct microbial community types within periodontitis-associated biofilms, which were associated with probing depth and clinical attachment loss. The relative abundance of *Tannerella forsythia* in diseased periodontal pockets was approximately 16- to 17-fold higher than that at healthy sites (Wirth et al., 2021). These findings support substantial ecological heterogeneity among periodontal pockets even within the same patient, rather than a uniform microbial composition across all advanced lesions.

A three-dimensional humanized gingival pocket model has further demonstrated that pocket-like anatomical architecture can generate oxygen gradients and support the simultaneous growth of aerobic, facultatively anaerobic, and obligately anaerobic bacteria, leading to biofilms with distinct spatial organization (Adelfio et al., 2023). However, this model was primarily inoculated with plaque derived from healthy individuals and lacked several key components of the *in vivo* periodontal environment, including immune cells, vasculature, bone tissue, and salivary flow. An exploratory study involving only eight patients found that, among deep periodontal pockets of comparable depth (6–10 mm), sites with higher local temperatures contained greater proportions of *P. gingivalis* and bacteria belonging to the red and orange complexes (Rams and Slots, 2024). Given the very small sample size, however, these findings should be regarded only as preliminary evidence that the local inflammatory microenvironment may exert selective pressure on microbial communities.

Microbial recovery after treatment does not necessarily occur in parallel with clinical improvement. A preliminary longitudinal study involving patients with periodontal disease and dental caries found that differences in the salivary microbiome and metabolome relative to healthy controls persisted for several months after clinical symptoms had improved (Yama et al., 2023). In another longitudinal 16S rRNA sequencing study of 38 patients with periodontitis, subgingival instrumentation induced a rapid shift in the microbiota toward a health-associated state at days 1 and 7; however, by day 90, some sites had re-established microbial communities resembling their pretreatment profiles (Johnston et al., 2023).

Further integrated metagenomic and metabolomic analyses have shown that the microbiota of patients with Stage III–IV periodontitis is characterized by enhanced functional potential related to lipopolysaccharide biosynthesis and bacterial motility, together with reduced short-chain fatty acid- and vitamin-related functions. Following nonsurgical periodontal therapy, both microbial community composition and metabolic output recovered only partially toward health-associated states (Baima et al., 2025b). Host exposure factors may also influence the extent of ecological recovery after treatment. In a 26-month follow-up study of 163 patients, higher exhaled carbon monoxide levels among smokers were associated with increased abundance of anaerobic communities dominated by Fusobacterium and Prevotella; however, self-reported smoking status explained microbial dysbiosis more strongly than carbon monoxide measurements themselves (Koehlen et al., 2026).

## 5 Microbiota-modulating therapeutic strategies for periodontitis and their translational potential

The development and progression of periodontitis result from complex interactions among oral microbial dysbiosis, dysregulated host immunity, and periodontal tissue destruction. With advances in our understanding of disease pathogenesis, therapeutic concepts have gradually shifted from a primary emphasis on reducing pathogenic microbial burden toward modulation of microbial community structure and function, with the broader goal of restoring microbiota–host homeostasis. Current microbiota-based therapeutic strategies mainly include conventional periodontal therapy and biofilm control, probiotics, prebiotics and dietary microbiota modulation, adjunctive anti-infective therapy, and precision microbiota-targeted interventions such as bacteriophages and engineered bacteria. Their major characteristics and recent research progress are summarized in Table 2.

**Table 2:** Microbiota-modulating therapeutic strategies for periodontitis and their translational status.

Therapeutic strategy	Proposed mechanism	Highest level of evidence	Human clinical evidence	Microbiome-related endpoints	Immune/inflammatory endpoints	Major limitations	Translational status	References
Conventional periodontal therapy and biofilm control	Mechanical disruption and removal of subgingival biofilms, reduction of microbial burden, and promotion of ecological recovery toward a health-associated community	Human longitudinal interventional studies	Established clinical benefit for PD, CAL, and BOP; longitudinal studies demonstrate treatment-associated shifts in oral microbial composition and function	Yes; community composition, microbial diversity, functional features, and recovery toward health-associated profiles	Limited/indirect; inflammation is primarily reflected by clinical periodontal improvement rather than comprehensive immune profiling	Deep periodontal biofilms may persist or recolonize; microbiome recovery is incomplete and heterogeneous among patients	Established standard of care	Baima et al. (2024); Simpson et al. (2024); Li L. et al. (2025)
Probiotic therapy	Competitive exclusion of periodontitis-associated microorganisms, production of antimicrobial metabolites, modulation of biofilm ecology, and potential regulation of local host immune responses	Randomized controlled clinical trials plus preclinical mechanistic studies	RCTs support possible adjunctive clinical benefits, although results are inconsistent across strains, regimens, and patient populations	Yes in selected trials; changes in oral/subgingival microbial composition and target taxa have been assessed	Partially assessed; anti-inflammatory and immunomodulatory effects are supported mainly by selected clinical and preclinical studies	Strong strain specificity, transient colonization, heterogeneous dosing and administration protocols, and uncertain long-term efficacy	Clinical adjunct under evaluation	Ranjith et al. (2022); de Oliveira et al. (2022); Gao et al. (2022)
Prebiotic and dietary modulation of the oral microbial ecosystem	Selective provision of substrates that favor health-associated microorganisms and metabolic functions, including nitrate-reducing pathways	Human dietary intervention studies plus preclinical mechanistic studies	Early human studies indicate that nitrate-rich dietary interventions can modify oral microbial composition; evidence for periodontal clinical benefit remains limited	Yes; microbial composition and nitrate-reduction capacity have been evaluated	Limited; direct immune endpoints are not consistently assessed	Evidence is concentrated on nitrate-based interventions; small study populations and limited long-term randomized trials restrict generalizability	Early clinical exploration	Mazurel et al. (2023); Jockel-Schneider et al. (2021); Rosier et al. (2024)
Local antimicrobial and adjunctive anti-infective therapy	Reduction of residual periodontitis-associated microorganisms and microbe-derived inflammatory stimulation through systemic or locally delivered antimicrobial approaches	Randomized placebo-controlled clinical trials	Adjunctive clinical benefits have been demonstrated in selected patient groups, with effects depending on antimicrobial agent, treatment duration, and disease phenotype	Yes; changes in target microorganisms, microbial community composition, and antimicrobial resistance profiles have been evaluated	Yes in selected trials; inflammatory and immunological outcomes have been assessed alongside clinical and microbiological responses	Potential disruption of commensal communities, variable patient benefit, antimicrobial resistance, and limited suitability for repeated or broad use	Clinically available as selective adjunctive therapy	Cosgarea et al. (2022); Cosgarea et al. (2021); Kang et al. (2021)
Bacteriophage therapy	Selective lysis or ecological suppression of target bacteria, including direct or indirect disruption of periodontitis-associated biofilms	Preclinical in vitro evidence	No established human clinical evidence for periodontitis	Yes in preclinical models; target bacterial suppression and biofilm-related outcomes	Limited; host immune consequences remain insufficiently characterized	Narrow host range, phage resistance or tolerance, delivery challenges within mature biofilms, ecological uncertainty, and unresolved safety issues	Preclinical / proof-of-concept	Wang Y. et al. (2022); Fujiki et al. (2021); Wu C. et al. (2024)
Engineered microorganisms and precision microbiota-targeted therapy	Selective depletion of disease-associated microorganisms or functional remodeling of microbial communities using microbiota transplantation, targeted microbial elimination, or bioengineered antimicrobial-producing bacteria	Animal intervention studies and preclinical proof-of-concept studies	No validated human clinical evidence for periodontitis	Yes in preclinical studies; target bacterial burden and/or microbial community restructuring have been assessed	Partially assessed; selected studies report inflammatory or tissue-destructive outcomes, but comprehensive immune validation is limited	Ecological stability, off-target effects, biosafety, controllability, durability, manufacturing, and regulatory requirements remain unresolved	Preclinical / experimental	Beikler et al. (2021); Yakar et al. (2024); Choudhury et al. (2025)

### 5.1 Conventional periodontal therapy and ecological control of biofilms

Periodontitis is a prototypical biofilm-associated disease, and controlling dysbiotic biofilm formation therefore remains the cornerstone of clinical management. Scaling and root planing (SRP) mechanically removes subgingival plaque and calculus, reduces the microbial burden within periodontal pockets, and limits persistent microbial stimulation. Extensive clinical evidence has demonstrated that SRP improves key periodontal parameters, including probing depth (PD), clinical attachment level (CAL), and bleeding on probing (BOP), and it remains a fundamental component of periodontitis treatment.

From an ecological perspective, SRP reduces the burden of subgingival biofilms through mechanical disruption, and longitudinal clinical studies further indicate that the microbial community can shift toward a more health-associated state after treatment (Baima et al., 2024; Simpson et al., 2024; Li L. et al., 2025). Periodontal biofilms, however, possess highly complex spatial organization and functional cooperation. Different microorganisms can maintain community stability through metabolic complementation, spatial coexistence, and intercellular communication, making complete eradication of mature biofilms difficult through mechanical debridement alone. This limitation is particularly relevant in deep periodontal pockets, where anaerobic communities may recolonize and potentially contribute to the persistence of local inflammation through continued microbe-associated stimulation (see Figure 3).

**Figure 3:**
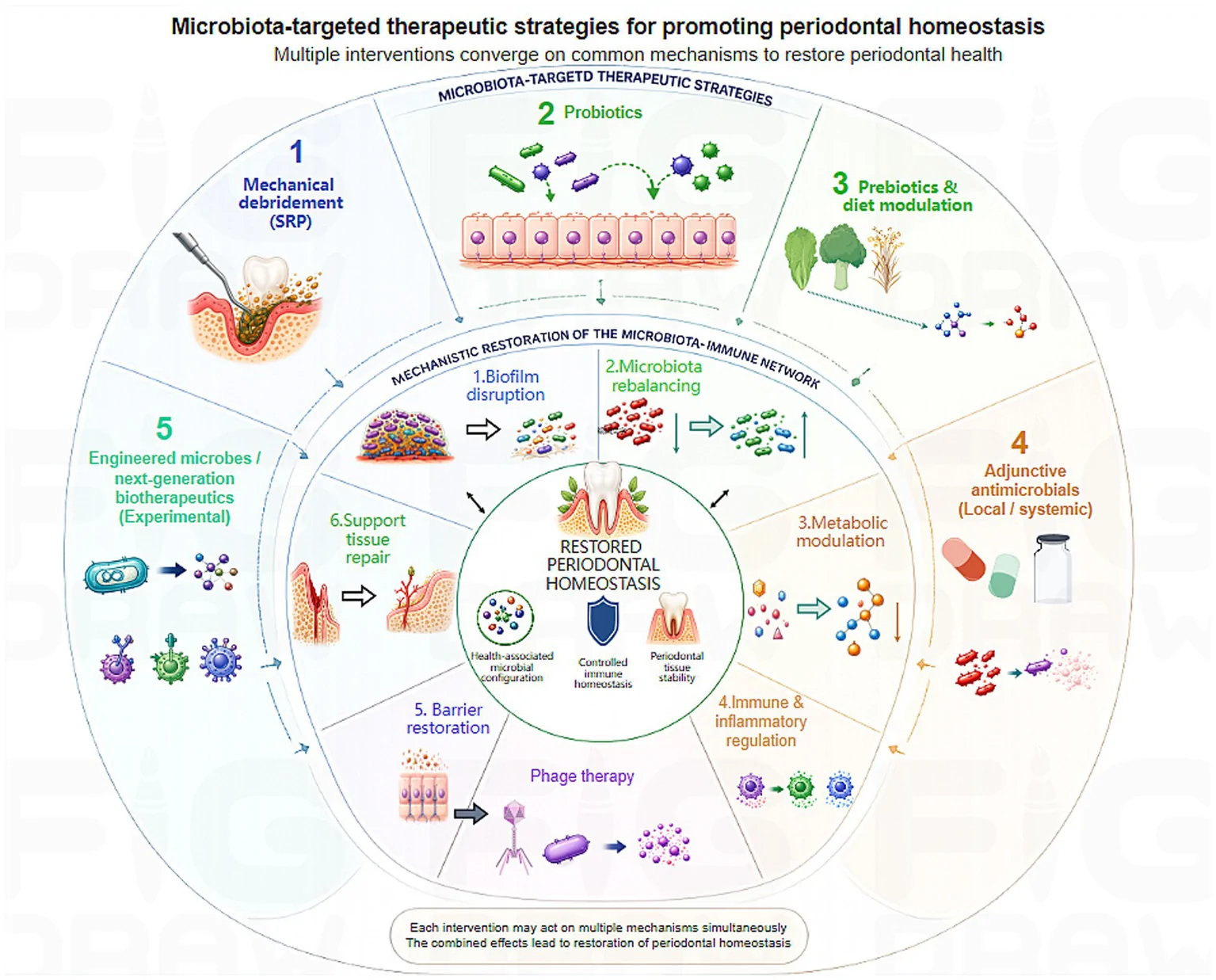
Microbiota-targeted therapeutic strategies for promoting periodontal homeostasis. This figure illustrates the conceptual framework of microbiota-targeted therapeutic interventions in periodontitis. Current and emerging strategies—including mechanical debridement, probiotics, prebiotics and dietary interventions, adjunctive antimicrobial therapy, bacteriophage-based approaches, and engineered microbes—collectively target multiple interconnected mechanisms, including biofilm disruption, microbiota rebalancing, metabolic modulation, immune and inflammatory regulation, barrier restoration, and tissue repair. Rather than acting through isolated pathways, these interventions converge on overlapping biological mechanisms to promote restoration of periodontal homeostasis. The translational maturity of each strategy is indicated using four categories: Clinical standard, Human clinical evidence, Early clinical/exploratory, and Preclinical/experimental. These categories reflect the highest level of currently available supporting evidence and are intended to distinguish established therapies from emerging or experimental approaches.

Clinical observations also indicate that disease recurrence may occur in some patients despite appropriate periodontal therapy, suggesting that disease persistence is determined not only by residual microbial burden but also by the local immune state and the extent to which a health-associated microbial ecosystem is re-established. An important future direction in periodontal therapy will therefore be to combine effective biofilm control with ecological interventions that actively promote restoration of a stable, health-associated oral microbiota.

### 5.2 Probiotic-mediated remodeling of the oral microbial ecosystem

Probiotics represent one of the microbiota-modulating approaches for periodontitis with a relatively substantial body of clinical evidence. Proposed mechanisms from experimental studies include competitive adhesion, competition for nutrients, and production of antimicrobial molecules, which may restrict the colonization of periodontitis-associated bacteria and interfere with biofilm formation. Probiotic-derived metabolites and immunomodulatory signals may also contribute to regulation of the local inflammatory microenvironment. To date, strains belonging to *Lactobacillus, Bifidobacterium*, and selected *Streptococcus* species have been investigated as adjunctive interventions for periodontitis.

Preclinical studies suggest that certain probiotics can inhibit the growth of periodontitis-associated bacteria such as *Porphyromonas gingivalis* and *Fusobacterium nucleatum* and reduce the expression of inflammatory mediators (Gao et al., 2022). Probiotics may also modulate the periodontal immune environment by influencing macrophage function, neutrophil responses, and the Th17/Treg balance. Clinical studies have reported that adjunctive probiotic therapy may provide additional improvements in PD, CAL, and BOP in some patients and may also alter the composition of the periodontal microbiota (Ranjith et al., 2022; de Oliveira et al., 2022). Nevertheless, findings remain heterogeneous across studies, likely because of differences in probiotic strains, dosage, routes of administration, and baseline microbial characteristics of the enrolled populations. Future studies integrating metagenomics and metabolomics to identify strains with reproducible ecological regulatory effects, together with microbiome-informed selection of probiotic interventions for individual patients, may facilitate the development of more precise and effective probiotic-based periodontal therapies.

### 5.3 Prebiotic and dietary strategies for modulating the oral microbial ecosystem

From theoretical and experimental perspectives, prebiotics may promote the growth of potentially beneficial microorganisms and modulate microbial metabolic functions by providing selectively utilized nutritional substrates. In periodontitis, prebiotics may exert beneficial effects by enhancing the competitive fitness of commensal bacteria, improving microbial metabolic profiles, and limiting the establishment of a pro-inflammatory microenvironment. Experimental and clinical studies, particularly those involving nitrate-based approaches, suggest that prebiotic and dietary modulation may influence oral microbial composition and metabolic activity (Mazurel et al., 2023; Jockel-Schneider et al., 2021; Rosier et al., 2024).

At present, research on prebiotics and diet-based microbiome modulation in periodontitis remains exploratory. Some studies suggest potential improvements in periodontal inflammatory parameters, but the overall evidence base is still limited. Importantly, substantial ecological and physiological differences exist between the oral cavity and the gastrointestinal tract, and experience derived from gut microbiota interventions therefore cannot be directly extrapolated to the oral environment. Future studies integrating oral metagenomics and metabolomics will be needed to define how specific nutritional interventions influence periodontal microbial communities, metabolic functions, and host immune states.

### 5.4 Adjunctive antimicrobial therapy and precision microbiota-targeted interventions

In addition to conventional periodontal therapy and microbiota modulation, adjunctive anti-infective treatment remains an important component of periodontitis management. Local antimicrobial agents and systemic antibiotics can reduce the burden of selected periodontitis-associated pathogens, decrease microbe-derived inflammatory stimulation, and provide additional clinical benefits in some patients with moderate-to-severe disease (Cosgarea et al., 2022; Cosgarea et al., 2021). However, periodontal microorganisms do not exist as isolated species but instead form complex communities characterized by extensive ecological interactions and functional interdependence.

In recent years, targeted microbiota interventions directed against specific microorganisms or functional pathways have attracted increasing attention. Because bacteriophages typically exhibit relatively high host specificity, a major theoretical advantage is their ability to reduce selected bacterial populations while minimizing disruption of non-target members of the microbiota. Current periodontitis-related bacteriophage research remains largely confined to *in vitro* proof-of-concept studies, which have demonstrated antimicrobial and antibiofilm potential (Wang Y. et al., 2022; Fujiki et al., 2021; Wu C. et al., 2024). Nevertheless, their efficacy *in vivo* and in humans, long-term effects on the overall oral microbial ecosystem, and consequences for host immune responses remain insufficiently characterized. Moreover, because periodontitis involves coordinated alterations across multiple microbial species and functional networks, a single bacteriophage is unlikely to adequately address the complexity of the pathological microbial ecosystem. Restricted host range, delivery efficiency, phage tolerance or resistance, ecological stability, and long-term safety therefore remain major challenges for clinical translation.

Emerging precision microbiota-targeted approaches include oral microbiota transplantation, targeted elimination of disease-associated microorganisms, and engineered microbial platforms (Beikler et al., 2021; Yakar et al., 2024; Choudhury et al., 2025). However, the supporting evidence differs substantially among these approaches. Oral microbiota transplantation has been evaluated in dogs with naturally occurring periodontitis (Beikler et al., 2021), whereas targeted elimination of *F. nucleatum* has been investigated in both an *in vitro* multispecies microbial community and a ligature-induced mouse periodontitis model (Yakar et al., 2024). In contrast, the bioengineered probiotic strategy reported in reference (Choudhury et al., 2025) was evaluated *in vitro* and in a simulated human gut microbiome in the context of colorectal cancer-associated *F. nucleatum*, rather than in a periodontal animal model. Therefore, its applicability to periodontitis remains to be directly validated.

However, it is important to distinguish target discovery from truly precision-guided clinical therapy. At present, metagenomics, metabolomics, and immunomics primarily enable the identification of microbial, metabolic, and immune features associated with disease states or treatment outcomes, which may facilitate patient stratification and the discovery of candidate therapeutic targets. Only when such features are independently validated and can reliably predict, before treatment, which patients are likely to benefit from a specific intervention can they be considered potential predictors of therapeutic response. True biomarker-guided therapy will require further demonstration that treatment selection based on prospectively validated molecular features improves patient outcomes compared with conventional treatment strategies.

Overall, microbiota-targeted therapy for periodontitis is expanding from simple reduction of microbial burden toward ecological modulation and functional targeting. SRP, biofilm control, and selected adjunctive antimicrobial and probiotic strategies already have a degree of clinical support, whereas prebiotics, bacteriophages, engineered bacteria, and other precision microbiota-targeted interventions remain at varying exploratory or preclinical stages. Future therapeutic strategies should aim not only to restore microbial ecological homeostasis but also to account for host immune status, tissue regenerative capacity, and interindividual variability, thereby enabling more stratified and empirically testable approaches to periodontal treatment.

## 6 Future perspectives

Oral microbiota–immune crosstalk provides an integrative framework for understanding the initiation and progression of periodontitis. In recent years, advances in microbiomics, metabolomics, immunomics, and single-cell technologies have increasingly revealed the complex relationships among microbial communities, the local metabolic milieu, and host immune states in periodontitis. These developments have also shifted the field from descriptive analyses of microbial composition toward investigation of microbial ecological function, host responses, and their bidirectional interactions. Nevertheless, substantial limitations remain in current evidence with respect to study design, technical standardization, causal inference, and clinical translation.

### 6.1 Current limitations and knowledge gaps

A large proportion of human studies remain cross-sectional in design. Although such studies can identify associations among microbial characteristics, immune states, and disease phenotypes, they are inherently limited in establishing temporal sequence and causal direction. Human longitudinal studies incorporating repeated sampling and long-term follow-up remain comparatively scarce. At the same time, the periodontal microbiota exhibits substantial geographic and interindividual variability, while age, smoking, glycemic status, oral hygiene, diet, medication use, and other systemic factors can further increase study heterogeneity. Differences in sample type, including subgingival plaque, saliva, gingival crevicular fluid, and gingival tissue, as well as variation in sampling sites and the selection of disease-associated locations, further limit direct comparability across studies.

At the technical level, sequencing platforms, bioinformatic pipelines, and multi-omics analytical approaches remain insufficiently standardized, creating challenges for cross-study integration of microbial composition, functional pathways, and metabolic signatures. Much of the available temporal and mechanistic evidence still relies on animal models such as ligature-induced periodontitis, which do not fully recapitulate the chronic, heterogeneous, and naturally evolving course of human disease. In addition, many studies examine microbial alterations or host immune changes separately, whereas relatively few simultaneously assess microbial communities, metabolic profiles, and immune endpoints. Consequently, it remains unresolved whether microbial dysbiosis primarily precedes immune dysfunction, results from it, or emerges through a dynamically self-reinforcing interaction between the two. For emerging microbiota-based interventions, including prebiotics, bacteriophages, engineered bacteria, and other targeted ecological strategies, clinical evidence in humans also remains limited, and their long-term efficacy, safety, and effects on the broader oral ecosystem require further evaluation.

### 6.2 Future research directions and precision translation

Future studies should place greater emphasis on longitudinal, multicenter, and standardized designs and integrate microbiomic, metabolomic, immunomic, spatial, and single-cell technologies to enable repeated dynamic assessment within the same individuals. Such approaches will be essential for more accurately resolving the temporal sequence of microbiota–immune interactions and identifying key regulatory nodes during disease progression. Host and environmental factors, including smoking, glycemic status, age, oral hygiene, diet, medication use, and systemic diseases, should also be incorporated into analytical frameworks to improve reproducibility and enhance the generalizability of patient-stratification models.

Importantly, identification of multi-omics signatures should not be equated with the achievement of precision therapy. At present, most studies remain at the stages of biomarker discovery, patient stratification, therapeutic target identification, and exploratory prediction of treatment response. True biomarker-guided clinical intervention will require independent validation of candidate signatures, assessment of their longitudinal stability, standardization of analytical procedures and decision thresholds, and demonstration that they provide incremental predictive or clinical decision-making value beyond established clinical parameters. Only when treatment selection based on prospectively validated molecular features leads to superior patient outcomes compared with conventional treatment strategies can multi-omics discoveries be considered successfully translated into precision clinical decision-making. Accordingly, precision treatment of periodontitis is currently better viewed as a future translational goal. By integrating microbial ecological features, host immune states, and treatment-response profiles, periodontal care may gradually move from broadly uniform interventions toward more stratified and individualized therapeutic decision-making.

## Glossary

AhR: aryl hydrocarbon receptor
BOP: bleeding on probing
C3: complement component 3
CAL: clinical attachment level
CD: cluster of differentiation
cGAS: cyclic GMP–AMP synthase
CYP1A1: cytochrome P450 family 1 subfamily A member 1
EPS: extracellular polymeric substances
GCF: gingival crevicular fluid
IFI16: interferon gamma-inducible protein 16
IgG: immunoglobulin G
IL: interleukin
ILC3: group 3 innate lymphoid cell
JAK: Janus kinase
JNK: c-Jun N-terminal kinase
LPS: lipopolysaccharide
MAMPs: microbe-associated molecular patterns
MAPK: mitogen-activated protein kinase
MIF: macrophage migration inhibitory factor
MMP: matrix metalloproteinase
MyD88: myeloid differentiation primary response 88
NETs: neutrophil extracellular traps
NF-κB: nuclear factor kappa B
NLRP3: NOD-like receptor family pyrin domain containing 3
NOD1: nucleotide-binding oligomerization domain-containing protein 1
Nrf2: nuclear factor erythroid 2-related factor 2
OMVs: outer membrane vesicles
OPG: osteoprotegerin
PD: probing depth
PDGF-BB: platelet-derived growth factor-BB
PDGFR-β: platelet-derived growth factor receptor beta
RANKL: receptor activator of nuclear factor-κB ligand
RELA: RELA proto-oncogene, NF-κB subunit (p65)
ROS: reactive oxygen species
SCFAs: short-chain fatty acids
SRP: scaling and root planing
STAT: signal transducer and activator of transcription
STING: stimulator of interferon genes
SUCNR1: succinate receptor 1
TBK1: TANK-binding kinase 1
Tfh: T follicular helper cell
Th17: T helper 17 cell
TLR: Toll-like receptor
TNF-α: tumor necrosis factor alpha
Treg: regulatory T cell
TRAP: tartrate-resistant acid phosphatase
